# Berberine in Inflammatory Bowel Disease: Integrative Regulation of the Microbiota–Immune–Barrier Axis

**DOI:** 10.3390/ijms27125220

**Published:** 2026-06-09

**Authors:** Junling Tang, Chuanhua Wang, Yang Yang, Xiangxiong Cheng, Siyu Chen, Shiwen Zhou, Jing Ma, Peimin Feng

**Affiliations:** 1 School of Clinical Medicine, Chengdu University of Traditional Chinese Medicine, Chengdu 610072, China; 17383310200@163.com (J.T.);; 2 Hospital of Chengdu University of Traditional Chinese Medicine, Chengdu 610072, China

**Keywords:** berberine, inflammatory bowel disease, gut microbiota, mucosal immunity, intestinal barrier, microbiota–immune–barrier axis, inflammation, drug delivery

## Abstract

Inflammatory bowel disease (IBD) is a chronic relapsing disorder associated with dysregulated interactions among the gut microbiota, mucosal immunity, and the intestinal barrier. Although current treatments have improved disease control, incomplete response, adverse effects, and relapse remain common. Berberine, a natural isoquinoline alkaloid, has gained attention as a multitarget compound with potential relevance to IBD. This narrative review summarizes evidence published up to March 2026 on the pharmacological basis, delivery optimization, clinical translation, and microbiota–immune–barrier axis regulation of berberine in IBD. Current evidence suggests that berberine may reshape gut microbial communities, regulate mucosal immune responses, and support epithelial barrier repair, thereby contributing to the restoration of intestinal homeostasis. Emerging formulation strategies, particularly microbiota-responsive and intestine-targeted delivery systems, may improve local exposure and strengthen its therapeutic potential. However, the current evidence is still dominated by preclinical studies. Clinical data remain limited, and the causal links among microbial remodeling, immune modulation, and barrier restoration are not yet fully defined. Future work should prioritize mechanistic validation, clinically relevant delivery design, and well-controlled clinical trials to clarify the role of berberine in personalized IBD management.

## 1. Introduction

Inflammatory bowel disease (IBD), comprising ulcerative colitis (UC) and Crohn’s disease (CD), is a group of chronic, relapsing inflammatory disorders of the gastrointestinal tract that imposes a growing global health burden [1,2,3]. Over recent decades, the incidence and prevalence of IBD have increased markedly, particularly in newly industrialized regions, likely reflecting changes in environmental exposures, dietary patterns, urbanization, and lifestyle [4,5,6,7,8]. Clinically, IBD is characterized by recurrent intestinal inflammation, abdominal pain, diarrhea, rectal bleeding, and progressive impairment in quality of life, and many patients require repeated hospitalization and long-term medical treatment [9,10]. Biologics and small-molecule agents have greatly improved IBD management. However, primary nonresponse, secondary loss of response, incomplete mucosal healing, and treatment-related adverse effects remain common clinical challenges [11]. These limitations underscore the need for therapeutic strategies that can better address the complex and relapsing nature of IBD.

The pathogenesis of IBD is multifactorial and involves a complex interplay among genetic susceptibility, environmental triggers, aberrant immune responses, and alterations in the gut microbial ecosystem [12,13]. These factors do not act independently but instead converge within an integrated microbiota–immune–barrier network that is central to intestinal homeostasis [14,15,16]. Under physiological conditions, commensal microbiota support immune education, metabolic balance, and epithelial maintenance. At the same time, the intestinal barrier limits excessive microbial translocation and prevents uncontrolled immune activation [17,18,19]. In IBD, this coordinated system is disrupted. Microbial dysbiosis can enhance inflammatory stimulation, mucosal immune activation can aggravate epithelial injury, and barrier dysfunction can further increase the translocation of microbial products and antigens. These events may reinforce one another and form a self-amplifying inflammatory circuit [20]. Therefore, the microbiota–immune–barrier axis provides not only a framework for understanding IBD pathogenesis, but also a basis for evaluating therapies that act on interconnected disease processes.

Current therapeutic approaches mainly target selected inflammatory mediators or signaling pathways, such as tumor necrosis factor-α (TNF-α), interleukins, and Janus kinase (JAK)-dependent cascades [21,22,23]. While these therapies have transformed the treatment landscape, their predominant focus on individual inflammatory nodes may not fully address the network-based pathophysiology of IBD, in which microbial imbalance, immune dysregulation, and epithelial injury are tightly interconnected [9,10,21]. As a result, there is increasing interest in therapeutic agents with broader regulatory capacity, particularly those able to influence several pathogenic processes in parallel and restore mucosal homeostasis [24]. Natural products derived from medicinal plants have attracted considerable attention in this context because of their multiple pharmacological activities, including anti-inflammatory, immunomodulatory, microbiota-regulating, and barrier-protective effects [25,26,27]. Such properties make them relevant candidates for evaluating therapeutic modulation of the microbiota–immune–barrier axis.

Berberine has attracted increasing attention for its therapeutic potential in inflammatory and metabolic disorders [28,29,30]. Experimental evidence suggests that berberine may alleviate intestinal inflammation through interconnected mechanisms, including suppression of inflammatory signaling, regulation of mucosal immune responses, restoration of epithelial barrier integrity, and remodeling of gut microbiota composition and function [31,32]. However, the causal order and relative contribution of these effects remain incompletely defined. For example, microbial changes after berberine treatment may reflect direct modulation of the gut ecosystem, secondary effects of reduced inflammation, or both. This uncertainty limits a coherent understanding of how berberine acts across the microbiota–immune–barrier axis.

Several reviews have examined berberine in the context of intestinal inflammation and ulcerative colitis. Habtemariam provided an early overview of the pharmacological rationale for using berberine in IBD [33]. Ashrafizadeh et al. summarized its anti-colitic effects, with emphasis on inflammation- and oxidative stress-related mechanisms [34]. Xiong et al. proposed a tuft cell-related pathway that may contribute to the therapeutic effects of berberine in UC [35]. More recently, Duda-Madej et al. reviewed the anti-inflammatory and gut microbiota modulatory effects of berberine in bowel health [36]. These studies have provided an important basis for understanding the intestinal actions of berberine, but the functional links among microbial regulation, microbial metabolites, mucosal immune modulation, and epithelial protection remain insufficiently integrated.

By contrast, this review frames berberine as a potential modulator of the microbiota–immune–barrier axis in IBD. By integrating microbiological, metabolic, immunological, and epithelial evidence, we evaluate whether its reported effects represent independent actions, sequential events, or mutually reinforcing processes. We also discuss delivery optimization, preclinical and clinical evidence, and key translational challenges, including model heterogeneity, limited human data, formulation design, local intestinal exposure, and dose conversion. This integrated perspective aims to provide a more coherent basis for understanding both the therapeutic potential and development challenges of berberine-based strategies in IBD.

## 2. Literature Search Strategy

This article was designed as a narrative review to summarize the mechanistic, translational, and therapeutic roles of berberine in inflammatory bowel disease (IBD), with particular emphasis on the microbiota–immune–barrier axis. Relevant studies published or available online up to March 2026 were searched in PubMed, Web of Science, and Scopus. Additional articles were identified through Google Scholar and manual screening of reference lists. The search terms included combinations of “berberine,” “inflammatory bowel disease,” “ulcerative colitis,” “Crohn’s disease,” “experimental colitis,” “gut microbiota,” “microbial metabolites,” “immune regulation,” “intestinal barrier,” “mucosal immunity,” “drug delivery,” and “colon-targeted delivery.”

Eligible publications included animal studies, in vitro mechanistic studies, microbiome- and metabolomics-related studies, pharmacological studies, drug delivery studies, available clinical studies, and relevant reviews. Priority was given to studies directly addressing berberine-mediated regulation of intestinal inflammation, gut microbial composition, microbial metabolites, mucosal immune responses, epithelial barrier function, or translational delivery strategies. Non-English articles, duplicate reports, conference abstracts without full text, and studies with limited relevance to IBD or intestinal inflammation were generally excluded. Because this was a narrative review, no formal risk-of-bias assessment or quantitative synthesis was performed. Instead, the included literature was critically organized according to mechanistic themes and translational relevance.

## 3. Pharmacological Basis of Berberine in Inflammatory Bowel Disease

### 3.1. Chemical Characteristics and Pharmacokinetic Features

Berberine is a protoberberine isoquinoline alkaloid widely distributed in medicinal plants such as *Coptis chinensis*, *Phellodendron amurense*, and *Berberis species* [37,38,39]. Its quaternary ammonium structure contributes to its distinctive physicochemical properties and broad biological activities. At the same time, this structure is associated with limited membrane permeability and inefficient intestinal absorption after oral administration [40,41].

Pharmacokinetically, berberine is characterized by low oral bioavailability. This is generally attributed to poor intestinal uptake, extensive first-pass metabolism, and active efflux mediated by transporters such as P-glycoprotein [42,43]. In systemic diseases, the low oral bioavailability of berberine is usually considered a pharmacokinetic limitation because it restricts circulating drug concentrations. In IBD, however, this feature may be therapeutically relevant. A substantial proportion of orally administered berberine may remain within the intestinal lumen or at the mucosal surface, where dysbiosis, immune activation, and epithelial barrier injury are central pathological events [31]. This gut-centered disposition allows berberine to interact with microbial communities, epithelial cells, and mucosal immune components. Experimental studies suggest that berberine regulates the intestinal microenvironment by reshaping microbial composition, modulating mucosal immune responses, and supporting epithelial barrier integrity [44,45]. Intestinal microorganisms can also transform berberine into metabolites with distinct absorption properties and biological activities [40,46]. However, the relative contributions of luminal, mucosal, and systemic mechanisms remain incompletely defined.

### 3.2. Therapeutic Implications in IBD

The therapeutic relevance of berberine in IBD is closely linked to the multifactorial nature of the disease. Rather than acting through a single dominant target, berberine appears to influence several disease-related processes within the intestinal microenvironment [47,48,49,50]. This profile is consistent with the current understanding of IBD as a disorder involving coordinated disruption of microbial ecology, mucosal immunity, and epithelial homeostasis.

Importantly, the multi-target pharmacology of berberine should be interpreted according to the strength of supporting evidence. Not all proposed targets or pathways have been validated by direct binding, enzymatic inhibition, or target-engagement assays. Therefore, the evidence supporting berberine-associated targets and pathways was classified in Supplementary Table S1 according to direct target evidence, in silico-supported evidence, cellular or in vivo pathway validation, and microbiota-mediated functional evidence [51,52]. Where available, representative quantitative or semi-quantitative data were included. For example, berberine has been reported to directly target NIMA-related kinase 7 (NEK7) and inhibit NEK7 activity with a half-maximal inhibitory concentration (IC50) of 4.2 μM, thereby blocking the NEK7–NOD-like receptor family pyrin domain-containing 3 (NLRP3) interaction and suppressing inflammasome activation. Other proposed mechanisms should be interpreted as pathway-level, cellular, in vivo, or microbiota-mediated evidence unless direct target-engagement data are available.

Broad biological activity also does not necessarily ensure consistent therapeutic efficacy. The low oral bioavailability and complex intestinal disposition of berberine may lead to variable local exposure, especially in inflamed intestinal tissues. These limitations have stimulated interest in formulation and delivery strategies aimed at improving intestinal retention, mucosal targeting, and site-specific drug release [53,54,55,56]. These strategies are discussed further in Section 5.

Taken together, the chemical structure and gut-centered pharmacokinetic behavior provide a pharmacological basis for its investigation in IBD. They also support the rationale for evaluating berberine through a microbiota–immune–barrier framework, which better reflects the gut-localized and multifactorial nature of its actions.

## 4. Integrative Regulation of the Microbiota–Immune–Barrier Axis by Berberine

Accumulating evidence suggests that the pathogenesis of IBD arises not from defects in a single system, but from disrupted crosstalk among the gut microbiota, mucosal immunity, and intestinal epithelial integrity [57,58,59]. In this pathological network, gut dysbiosis, persistent immune hyperactivation, and epithelial barrier disruption act synergistically to fuel a self-amplifying cycle of chronic intestinal inflammation [60,61,62]. Building on this framework, berberine appears to exert pleiotropic and integrative effects across multiple interconnected layers of the intestinal microenvironment, including gut microbial structure, microbiota-derived metabolic signals, mucosal immune responses, and epithelial barrier function.

**Figure 1:**
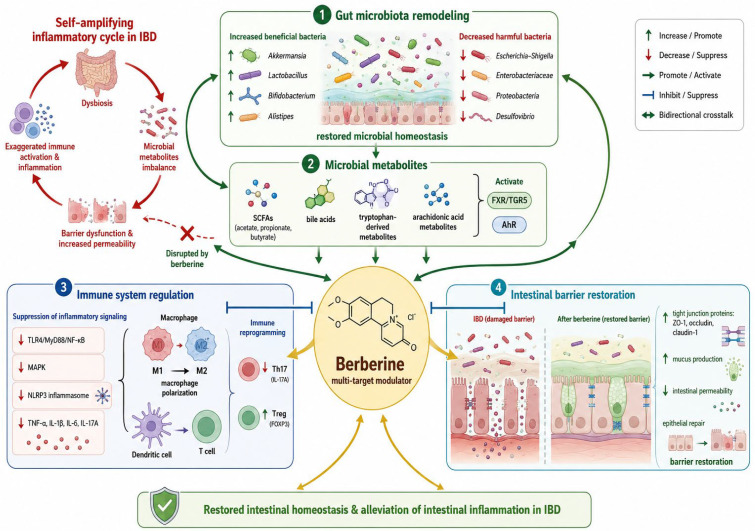
Berberine regulates the microbiota–immune–barrier axis in inflammatory bowel disease (IBD). Berberine acts as a multitarget modulator that reshapes gut microbiota, regulates microbiota-derived metabolites, suppresses mucosal immune inflammation, and restores intestinal epithelial barrier integrity. Through coordinated regulation of these interconnected processes, berberine helps interrupt the self-amplifying inflammatory cycle in IBD and promotes the recovery of intestinal homeostasis.

This microbiota–immune–barrier regulatory framework is schematically summarized in Figure 1. The following sections examine how berberine acts on individual components of this axis and how these effects may interact to restore intestinal homeostasis.

### 4.1. Regulation of Gut Microbiota and Microbiota-Derived Metabolites by Berberine

Gut dysbiosis is a common feature of IBD [63,64]. Active IBD is often accompanied by reduced microbial diversity, depletion of beneficial commensals, and expansion of pathobionts or inflammation-associated taxa. Although microbial signatures differ among disease phenotypes, host backgrounds, and analytical approaches, accumulating evidence supports a contributory role of dysbiosis in IBD pathogenesis, rather than viewing it only as a consequence of inflammation. In this context, the interaction between berberine and the gut microbiota is particularly relevant. Berberine should not be regarded only as a broad-spectrum antimicrobial compound. It can also act as a regulator of intestinal microbial ecology. The gut microbiota may serve not only as a target of berberine, but also as a mediator linking berberine exposure to its anti-inflammatory and barrier-protective effects.

#### 4.1.1. Remodeling of Gut Microbial Composition

Multiple studies have shown that berberine can partially reverse gut dysbiosis in experimental models of IBD. This effect is reflected by changes in microbial diversity and community structure, including alterations in α-diversity, β-diversity, and the Firmicutes/Bacteroidetes ratio [65,66,67,68]. These findings suggest that berberine does not act only on individual bacterial taxa but may reshape the intestinal microbial ecosystem at a broader community level. However, global ecological indices alone provide limited mechanistic information. Evidence from microbiota-depletion and microbiota-transfer experiments further supports the involvement of gut microbiota in the protective effects of berberine. Antibiotic-mediated depletion of intestinal microbiota markedly weakens the anti-inflammatory, barrier-protective, and mucosal-healing effects of berberine. Conversely, fecal microbiota transplantation from berberine-treated donors can reproduce several protective phenotypes in recipient models [69,70,71]. These findings indicate that gut microbiota is not only altered during berberine treatment but also participates in mediating its therapeutic effects.

#### 4.1.2. Modulation of Beneficial Commensals and Pro-Inflammatory Pathobionts

At the taxonomic level, berberine appears to reshape the gut microbiota through a bidirectional pattern: enrichment of beneficial or homeostasis-associated commensals and reduction in inflammation-associated pathobionts. Berberine has been reported to increase bacterial taxa associated with mucosal protection and microbial stability, while reducing taxa linked to dysbiosis, endotoxin burden, oxidative stress, and intestinal inflammatory injury [72,73].

Several beneficial or inflammation-negative taxa are enriched following berberine treatment, including *Akkermansia, Bacteroides, Lactobacillus, Bifidobacterium, Alistipes*, members of the Muribaculaceae family, and *Dubosiella* [65,66,74,75,76,77,78,79,80]. In contrast, berberine reduces the abundance of dysbiosis- or inflammation-associated taxa, such as *Escherichia–Shigella*, Enterobacteriaceae, *Desulfovibrio*, and Proteobacteria [70,73,80,81,82,83]. Together, these microbial changes suggest a shift from a pro-inflammatory microbial configuration toward a more protective and homeostasis-associated profile.

Nevertheless, these findings should be interpreted cautiously because microbial responses to berberine may vary according to animal species, colitis models, sequencing methods, dosage, treatment duration, and baseline microbiota composition. In addition, whether these taxonomic changes directly drive therapeutic outcomes or occur secondarily after inflammation is reduced remains to be clarified.

#### 4.1.3. Regulation of Microbiota-Dependent Metabolic Pathways

Berberine-induced alterations in the gut microbiota are accompanied by functional changes in microbiota-associated metabolic pathways, which may serve as an important mechanistic bridge between microbial remodeling and host protection [84,85,86,87,88]. To date, research has primarily focused on short-chain fatty acids (SCFAs), bile acid metabolism, tryptophan-related pathways, and arachidonic acid (AA) metabolism. However, evidence linking individual metabolic pathways to specific anti-inflammatory or barrier-protective outcomes remains fragmented and requires further integration.

Short-chain fatty acids (SCFAs), including acetate, propionate, and butyrate, play essential roles in epithelial energy metabolism, intestinal barrier integrity, and mucosal immune regulation. In patients with ulcerative colitis, reduced SCFA levels are frequently associated with impaired epithelial function, increased intestinal permeability, and compromised immune tolerance. A growing body of research has shown that berberine increases the abundance of SCFA-producing bacteria. These changes may help restore intestinal SCFA availability and improve the metabolic microenvironment of the colonic mucosa [89,90].

Beyond SCFA metabolism, berberine also regulates bile acid homeostasis and tryptophan metabolism, while modulating arachidonic acid (AA) pathways. Emerging data indicate that berberine can alter the bile acid profile and activate farnesoid X receptor (FXR)- and G protein-coupled bile acid receptor 1 (TGR5)-mediated signaling pathways [91]. In addition, berberine has been shown to regulate tryptophan metabolism and aryl hydrocarbon receptor (AhR)-related signaling [92], as well as suppress AA metabolism and the production of pro-inflammatory mediators such as prostaglandins and leukotrienes [76,93]. These pathways are closely related to epithelial barrier maintenance, immune tolerance, and resolution of inflammatory responses, suggesting that microbial metabolic remodeling may contribute to the downstream protective effects of berberine.

Taken together, berberine-mediated regulation of the gut microbiota extends beyond compositional remodeling to include functional modulation of microbiota-derived metabolic outputs. This microbial–metabolic remodeling may provide an upstream basis for the immunoregulatory and barrier-protective effects discussed in the following sections. Nevertheless, whether these metabolic alterations directly drive therapeutic outcomes or occur secondarily after inflammation is reduced remains to be further clarified.

### 4.2. Modulation of Intestinal Immune Responses

**Figure 2:**
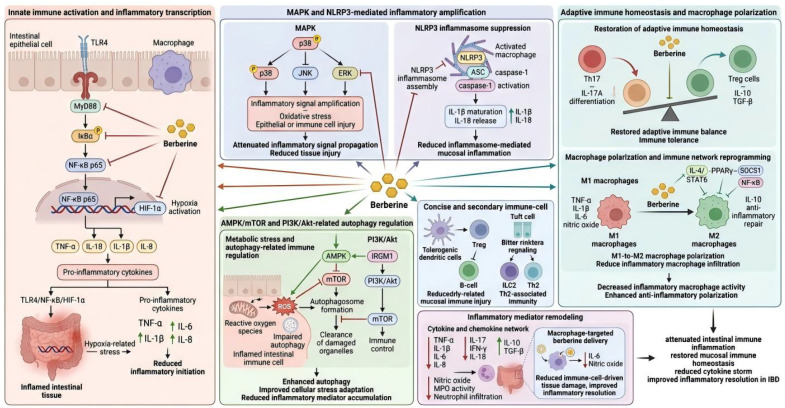
Molecular signaling network underlying the immunomodulatory effects of berberine in inflammatory bowel disease. Berberine alleviates intestinal mucosal inflammation by coordinately suppressing TLR4/MyD88/NF-κB/HIF-1α, MAPK, and NLRP3 inflammasome signaling, while modulating AMPK/mTOR- and PI3K/Akt-related autophagy pathways. These effects reduce pro-inflammatory mediator production, restore Th17/Treg balance, promote M2 macrophage polarization, and ultimately support mucosal immune homeostasis and inflammatory resolution in IBD.In the figure, green upward arrows indicate increased levels or activation, whereas red downward arrows indicate decreased levels or inhibition. Abbreviations: IBD, inflammatory bowel disease; TLR4, Toll-like receptor 4; MyD88, myeloid differentiation primary response 88; NF-κB, nuclear factor kappa B; HIF-1α, hypoxia-inducible factor-1 alpha; MAPK, mitogen-activated protein kinase; NLRP3, NOD-like receptor family pyrin domain-containing 3; AMPK, AMP-activated protein kinase; mTOR, mechanistic target of rapamycin; PI3K, phosphoinositide 3-kinase; Akt, protein kinase B; Th17, T helper 17; Treg, regulatory T cell.

Immune dysregulation is a central driver of chronic mucosal inflammation in inflammatory bowel disease (IBD). Available preclinical evidence indicates that berberine exerts immunomodulatory effects by acting on innate immune activation, adaptive immune imbalance, and the local inflammatory mediator network [34,94,95,96]. These effects are closely linked to the microbiota–immune–barrier axis, because microbial signals, epithelial injury, and immune activation mutually reinforce each other during intestinal inflammation. Therefore, the immunoregulatory activity of berberine should be understood as part of an integrated mucosal regulatory network rather than as inhibition of a single inflammatory pathway. The major intracellular signaling pathways underlying the immunoregulatory and barrier-protective effects of berberine are summarized in Figure 2.

#### 4.2.1. Suppression of Innate Immune Activation and Inflammatory Signaling

The innate immune system is the first line of intestinal defense and plays a central role in the initiation of mucosal inflammation in IBD. Persistent activation of innate immune pathways promotes excessive cytokine production and sustained tissue injury [30,47,97,98]. To clarify the functional relationships among the signaling pathways involved, these mechanisms can be organized into three interconnected modules: inflammatory initiation and transcriptional activation, inflammatory amplification and inflammasome activation, and metabolic stress- or autophagy-related regulation.

The first module involves inflammatory initiation and transcriptional activation. Pattern-recognition receptor signaling, particularly the TLR4/MyD88/NF-κB axis, is a key upstream driver of innate immune activation in intestinal inflammation [95,99,100]. Activation of this pathway promotes the transcription of pro-inflammatory mediators, including TNF-α, IL-1β, IL-6, and IL-8. Berberine has been reported to suppress NF-κB activation by reducing p65 nuclear translocation and IκBα phosphorylation, thereby decreasing NF-κB-dependent cytokine production and alleviating colonic injury [101,102,103]. In addition, berberine interferes with the TLR4/NF-κB/HIF-1α axis, suggesting a link between inflammatory transcription and hypoxia-related stress responses in inflamed intestinal tissues [104]. Thus, regulation of NF-κB-related signaling represents an important mechanism by which berberine limits the initiation of mucosal inflammation.

The second module involves inflammatory amplification and inflammasome activation. In parallel with NF-κB signaling, the MAPK pathway, including p38, JNK, and ERK, contributes to the propagation of inflammatory signals, oxidative stress, and epithelial injury. Berberine has been shown to reduce MAPK phosphorylation in experimental colitis models, which may attenuate inflammatory signal amplification and epithelial apoptosis [101,102,105]. Beyond transcriptional regulation, berberine also acts on inflammasome-mediated inflammatory responses. The NLRP3 inflammasome promotes caspase-1-dependent maturation and release of IL-1β and IL-18, thereby sustaining mucosal inflammation. Berberine inhibits NLRP3 inflammasome activation, reduces caspase-1 activity, and suppresses IL-1β and IL-18 release in models of intestinal inflammation [47,97,98]. These findings indicate that MAPK and NLRP3-related pathways are not isolated targets but are involved in limiting the amplification and persistence of innate immune responses.

The third module involves metabolic stress and autophagy-related regulation. In inflamed intestinal tissues, immune activation is closely linked to metabolic stress, mitochondrial dysfunction, and impaired autophagy. Berberine activates AMP-activated protein kinase (AMPK) and inhibits mechanistic target of rapamycin (mTOR) signaling, which may promote autophagy, enhance the clearance of damaged organelles, and reduce the accumulation of inflammatory mediators [106,107,108]. Recent evidence further suggests that immunity-related GTPase family M protein 1 (IRGM1) may be a potential target of berberine. Modulation of the IRGM1–PI3K/Akt/mTOR axis may therefore provide an additional connection between autophagy regulation and mucosal immune control [106,108]. Because AMPK/mTOR and PI3K/Akt/mTOR signaling interact with NF-κB and MAPK pathways, this module may indirectly restrain innate immune activation while improving cellular stress adaptation.

Taken together, berberine suppresses innate immune activation through coordinated regulation of inflammatory and metabolic signaling networks. NF-κB-related signaling mainly contributes to inflammatory initiation and cytokine transcription. MAPK and NLRP3 pathways participate in inflammatory amplification and inflammasome activation. AMPK/mTOR-, PI3K/Akt/mTOR-, and IRGM1-related pathways are more closely associated with metabolic stress responses and autophagy control. This modular interpretation avoids presenting these pathways as a simple list of independent targets and better explains how berberine may coordinate innate immune suppression with mucosal homeostasis.

#### 4.2.2. Restoration of Adaptive Immune Homeostasis

In addition to restraining innate immunity, berberine corrects adaptive immune dysregulation characteristic of IBD. A hallmark of disease pathogenesis is the imbalance between pro-inflammatory T helper 17 (Th17) cells and immunosuppressive regulatory T (Treg) cells [97,109,110,111]. Accumulating evidence indicates that berberine suppresses Th17 differentiation and interleukin-17A (IL-17A) production while promoting Treg expansion and function, thereby restoring immune balance [107,109,111,112]. This shift is accompanied by coordinated transcriptomic reprogramming, characterized by downregulation of pro-inflammatory genes and upregulation of immunoregulatory pathways, ultimately redirecting the intestinal immune milieu toward inflammation resolution and tissue repair [94,97,109].

Berberine may also modulate B-cell activation and antibody production, further contributing to the attenuation of mucosal immune injury [113]. Macrophage polarization represents a critical interface linking innate and adaptive immunity. Berberine promotes the transition from pro-inflammatory M1 macrophages to anti-inflammatory M2 phenotypes, accompanied by decreased TNF-α and IL-1β and increased IL-10 production [109,114,115,116]. This phenotypic reprogramming is mediated by multiple signaling pathways, including IL-4/signal transducer and activator of transcription 6 (STAT6), AMPK-PPARγ, and AKT1/suppressor of cytokine signaling 1 (SOCS1)/NF-κB [30,114,116]. In macrophage–organoid co-culture systems, berberine effectively suppresses M1 macrophage infiltration and disrupts pathological macrophage–epithelial interactions [104,117].

Furthermore, berberine promotes the generation of tolerogenic dendritic cells and enhances Treg differentiation [118,119]. Emerging evidence suggests that berberine also modulates innate lymphoid cell responses via tuft cell-mediated signaling and bitter taste receptor pathways, influencing group 2 innate lymphoid cells (ILC2) and T helper 2 (Th2)-associated immunity [35,120,121].

Collectively, these findings indicate that berberine regulates adaptive immunity through coordinated immune network reprogramming rather than isolated modulation of individual cell populations.

#### 4.2.3. Remodeling of the Inflammatory Mediator Network

By broadly modulating cytokine and chemokine expression, berberine reshapes the immune microenvironment in inflamed tissues. It consistently suppresses key pro-inflammatory mediators, including TNF-α, IL-1β, IL-6, IL-17, and interferon-γ (IFN-γ), reflecting a systemic attenuation of the inflammatory cytokine network [122,123,124,125]. Concurrently, berberine enhances anti-inflammatory mediators such as IL-10 and TGF-β and reduces myeloperoxidase (MPO) activity and neutrophil infiltration, thereby limiting immune cell–driven tissue damage [122,123,124,125]. Recent advances in delivery systems further highlight the importance of targeted immunomodulation. Macrophage-directed delivery of berberine has been shown to more effectively suppress IL-6 and nitric oxide production while increasing IL-10 levels, leading to improved histological repair of inflamed tissues [115,124].

Taken together, berberine-mediated immune regulation in IBD involves suppression of inflammatory signaling, rebalancing of mucosal immune responses, and attenuation of cytokine-driven inflammatory amplification. These effects are closely linked to microbial remodeling and epithelial barrier repair within the microbiota–immune–barrier axis. Thus, mucosal barrier restoration should be viewed not only as a downstream consequence of reduced inflammation but also as a functional readout of coordinated microbial and immune regulation mediated by berberine [94,126,127]. Nevertheless, because most immune-related evidence is derived from experimental colitis models and cell-based systems, these findings should be interpreted as evidence of immunomodulatory activity rather than definitive proof of direct molecular target engagement.

### 4.3. Protection of Intestinal Mucosal Barrier Homeostasis

Within the microbiota–immune–barrier axis, the intestinal mucosal barrier is not only the physical foundation of intestinal homeostasis. It is also a dynamic interface that integrates microbial signals and host immune responses. Evidence indicates that berberine protects the mucosal barrier through several related mechanisms. These include restoration of tight junctions, preservation of the mucus layer, reduction in epithelial stress injury, and promotion of mucosal repair [128,129,130].

#### 4.3.1. Restoration of Mechanical Barrier Integrity

The mechanical barrier is composed of intestinal epithelial cells and intercellular junctional complexes. In IBD, inflammatory stimuli often reduce or mislocalize tight junction proteins, including zonula occludens-1 (ZO-1), occludin, claudin-1, and junctional adhesion molecule-A (JAM-A). This disruption increases paracellular permeability. Multiple studies have shown that berberine upregulates these proteins, stabilizes intercellular junctions, and reduces pathological intestinal permeability [131,132]. Optimized delivery systems further support these barrier-protective effects. Colon-targeted microspheres, hydrogels, and self-assembled nanoparticles can increase local drug accumulation at colonic lesions. This may improve the efficiency of epithelial barrier repair [133,134]. Mechanistically, berberine appears to preserve tight junction integrity through pathways involved in junctional organization and cytoskeletal regulation. These include HSP90AA1/MAPK14, QPCT-PI3K-Akt/MAPK, and AMPK/myosin light-chain kinase (MLCK) signaling [101,127,135]. Among these mechanisms, swiprosin-1 has been identified as a potential regulatory hub of the AMPK/MLCK pathway. This pathway may help prevent tight junction disassembly under inflammatory stress [101,127,135]. Together, these mechanisms converge on the stabilization of tight junction dynamics and epithelial integrity.

#### 4.3.2. Preservation of the Mucus and Chemical Barrier

The mucus layer secreted by goblet cells separates luminal microorganisms from the epithelial surface. In IBD, goblet cell depletion and mucus thinning weaken this protective layer. As a result, luminal antigens and microorganisms are more likely to contact the epithelium directly. This process can further amplify barrier injury and mucosal inflammation. Berberine has been shown to reverse dextran sulfate sodium (DSS)-induced goblet cell loss and mucus degradation [44,83,129]. Mechanistic studies further indicate that berberine upregulates Muc2 expression and supports mucin function. These effects help restore the surface chemical barrier of the injured epithelium [83,90,136].

#### 4.3.3. Inhibition of Epithelial Stress Injury and Apoptosis

Beyond structural disruption, epithelial stress and excessive apoptosis directly contribute to barrier breakdown. Berberine alleviates endoplasmic reticulum stress in colitis models. It also downregulates stress- and apoptosis-related molecules, including glucose-regulated protein 78 (GRP78), caspase-12, and caspase-3. These effects may enhance intestinal epithelial cell survival under inflammatory conditions [129,137]. In cellular injury models and organoid systems, berberine protects epithelial cells against inflammatory toxicity and preserves tight junction integrity [104,128]. It also reduces excessive reactive oxygen species (ROS) production and limits stress signal amplification through receptor-mediated pathways, including TAS2R38, AhR, and FXR [94,126,138]. These findings suggest that berberine protects the epithelial barrier not only by restoring junctional proteins, but also by improving epithelial stress tolerance. Notably, some in vitro studies have reported that high-dose berberine may induce transient tight junction internalization and increase permeability in non-inflamed intestinal epithelial models [132]. This observation suggests that the barrier effects of berberine may depend on dose and pathological context. Therefore, its barrier-protective effects under inflammatory conditions should be distinguished from potential stimulatory effects under physiological conditions.

#### 4.3.4. Promotion of Mucosal Repair and Barrier Reconstruction

Barrier protection by berberine is not limited to preventing epithelial damage. It also involves mucosal repair and tissue reconstruction. Berberine improves colonic crypt disorganization, restores epithelial integrity, and accelerates mucosal healing. These effects have been associated with regenerative pathways such as HIF-1, Wnt/β-catenin, and IL-22 signaling [71,139]. Organoid co-culture studies further show that berberine can normalize pathological crosstalk between epithelial cells and surrounding stromal or immune cells [104,117]. This may create a microenvironment that supports stem cell differentiation and mucosal reconstruction. Together, these findings suggest that berberine contributes to barrier recovery by promoting both epithelial repair and tissue-level remodeling.

Collectively, these findings support a coordinated model of berberine-mediated barrier protection. In this model, berberine reinforces epithelial structural integrity, enhances mucus-associated chemical defense, improves epithelial stress resistance, and promotes regenerative repair. These barrier-directed effects not only contribute to intestinal mucosal homeostasis but also provide a functional interface through which microbial remodeling and immune regulation converge within the microbiota–immune–barrier axis.

### 4.4. Integrated Crosstalk Within the Microbiota–Immune–Barrier Axis

The therapeutic significance of berberine in IBD is best understood within the microbiota–immune–barrier axis rather than through its effects on individual compartments in isolation [79,140,141]. In IBD, microbial dysbiosis, persistent immune activation, and epithelial barrier disruption amplify one another and jointly sustain chronic intestinal inflammation. Within this interconnected network, berberine may help restore intestinal homeostasis through combined effects on gut microbial remodeling, mucosal inflammatory restraint, and epithelial barrier support [33,36,78,142].

At the microbial level, berberine-mediated remodeling of the gut microbiota may reduce pro-inflammatory luminal stimuli and restore beneficial microbial metabolites, including SCFAs, bile acids, and tryptophan-derived metabolites [74,89]. These microbial and metabolic changes may contribute to immune restraint and epithelial support. Conversely, attenuation of mucosal immune activation may reduce cytokine-driven tissue injury, thereby creating a local environment more favorable for epithelial recovery and microbial re-equilibration [70,92,143,144]. Barrier restoration also plays a central role in this regulatory network. Improved epithelial integrity and reduced intestinal permeability may limit the translocation of luminal antigens, endotoxins, and microorganisms into the lamina propria, thereby reducing persistent immune stimulation [71,130,145]. A restored epithelial and mucus barrier may further provide a more stable ecological niche for beneficial commensals [135].

However, current evidence does not establish a definitive linear causal sequence among microbiota remodeling, immune regulation, and epithelial barrier repair. Several non-mutually exclusive models should therefore be considered. In a microbiota-first model, berberine-induced changes in microbial composition and microbial metabolites may precede immune recalibration and epithelial repair. In an immune-first model, suppression of inflammatory signaling and cytokine production may secondarily improve the epithelial niche and microbial ecology. In a barrier-first model, restoration of epithelial integrity and reduced permeability may decrease microbial translocation and downstream immune activation. Alternatively, given berberine’s limited systemic bioavailability but relatively high intestinal exposure, these effects may occur in parallel within the gut lumen and mucosa rather than through a single initiating event.

Taken together, the microbiota–immune–barrier axis provides a useful working model for understanding the broad protective effects of berberine in IBD [85,146,147]. Nevertheless, this model should not yet be interpreted as a confirmed causal pathway, because most available studies rely on endpoint observations in experimental colitis models, in vitro inflammatory systems, or microbiota/metabolite correlation analyses. Future studies using longitudinal sampling, microbiota-depleted or germ-free models, fecal microbiota transplantation, metabolite rescue experiments, cell-specific pathway inhibition, and time-resolved multi-omics analyses are needed to define the temporal hierarchy and causal directionality of berberine-mediated intestinal protection.

## 5. Delivery Strategies for Enhancing Gut-Localized Berberine Activity in Inflammatory Bowel Disease

Although berberine shows anti-inflammatory, immunoregulatory, microbiota-modulating, and barrier-protective effects in experimental IBD models, its translational application is limited by poor solubility, low membrane permeability, rapid elimination, and insufficient accumulation at inflamed intestinal sites. In IBD, mucus alteration, inflammatory exudation, accelerated intestinal transit, epithelial disruption, and lesion heterogeneity may further reduce effective mucosal exposure. Therefore, delivery optimization should prioritize gut-localized activity rather than systemic bioavailability.

**Figure 3:**
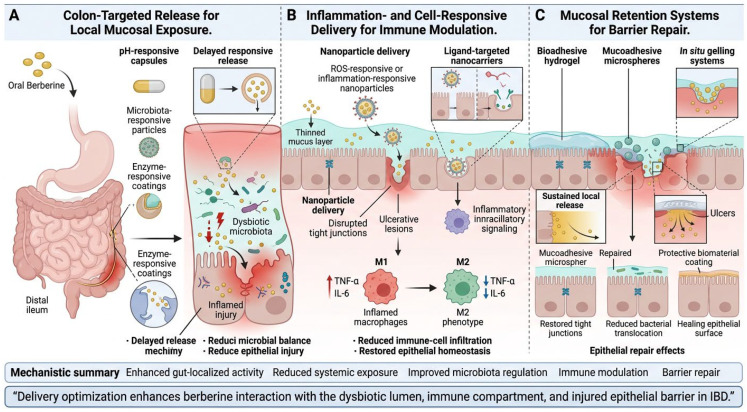
Delivery strategies for enhancing gut-localized berberine activity in inflammatory bowel disease. Berberine delivery systems may improve local intestinal exposure through three main strategies: colon-targeted release to increase drug availability in the dysbiotic lumen, inflammation- or cell-responsive delivery to enhance interaction with mucosal immune and epithelial cells, and mucosal retention systems to prolong drug contact with injured epithelial surfaces. These approaches aim to strengthen the local engagement of berberine with the microbiota–immune–barrier axis while reducing premature release and insufficient accumulation in inflamed intestinal tissues. In the figure, red upward arrows indicate increased levels, whereas green downward arrows indicate decreased levels or inhibition.

In this context, delivery systems are not merely formulation improvements, but a means to better align berberine exposure with the microbiota–immune–barrier axis. Their translational value lies in enhancing local drug engagement at three disease-relevant interfaces: the dysbiotic intestinal lumen, the immune–epithelial interface, and the injured epithelial barrier. Accordingly, this section summarizes three functional delivery strategies—colon-targeted release, inflammation- or cell-responsive delivery, and mucosal retention systems—which are illustrated in Figure 3 and summarized in Table 1.

### 5.1. Colon-Targeted Release for Local Mucosal Exposure

Colon-targeted release aims to increase berberine availability in intestinal regions affected by IBD. Conventional formulations may release berberine prematurely in the stomach or proximal small intestine, thereby reducing delivery to the distal ileum and colon. pH-responsive, enzyme-responsive, and microbiota-responsive systems have therefore been developed to delay drug release until the colonic environment is reached [55,66,73,131,149]. By increasing berberine exposure in the dysbiotic lumen and near the mucosal surface, these systems may strengthen its engagement with microbial imbalance, inflammatory signaling, and epithelial injury.

However, the performance of colon-targeted systems may vary under IBD conditions, where intestinal pH, transit time, microbial enzyme activity, mucus properties, and lesion distribution are often altered. Therefore, their value should be assessed not only by in vitro release profiles, but also by disease-site accumulation and mucosal exposure in inflammation-relevant models.

### 5.2. Inflammation- and Cell-Responsive Delivery for Immune Modulation

After reaching the inflamed intestine, berberine must access key mucosal targets, especially epithelial cells and inflammatory macrophages. Nanoparticle-based systems have been used to improve berberine dispersibility, mucus penetration, epithelial uptake, and immune-cell delivery [56,67,115,121,134]. These systems may amplify the local immunomodulatory effects of berberine at the immune–epithelial interface. Some macrophage-targeted carriers may also promote M1-to-M2 macrophage polarization [56,115].

Nevertheless, evidence for inflammation- or immune-cell-targeted delivery remains largely preclinical. Whether these systems can achieve selective and reproducible targeting in the heterogeneous mucosal environment of human IBD requires further validation.

### 5.3. Mucosal Retention Systems for Barrier Repair

Barrier injury is a central feature of IBD. Free berberine may be rapidly diluted or cleared from ulcerated mucosa by luminal fluid, inflammatory exudates, and accelerated transit. Bioadhesive hydrogels, in situ gelling systems, mucoadhesive microspheres, and polysaccharide-based carriers have therefore been designed to prolong local exposure and provide sustained release at injured mucosal sites [87,128,131,144,148]. By extending berberine contact with the epithelial repair interface, these systems may support tight-junction restoration, mucus layer repair, and reduced bacterial translocation.

However, mucosal retention must be carefully balanced. Excessive adhesion or prolonged retention may interfere with mucus clearance or epithelial renewal, particularly in ulcerated or highly inflamed mucosal regions.

### 5.4. Translational Relevance and Current Limitations

Overall, delivery optimization may improve the alignment between berberine exposure and the microbiota–immune–barrier axis by enhancing local drug engagement at the dysbiotic lumen, immune–epithelial interface, and injured epithelial barrier. Its main translational value lies in improving gut-localized activity rather than systemic bioavailability. However, whether these formulation advantages translate into sustained therapeutic benefit in human IBD remains unclear.

Current evidence is still dominated by in vitro systems and acute chemically induced colitis models, especially DSS- or 2,4,6-trinitrobenzene sulfonic acid (TNBS)-induced models. These models are useful for early validation but do not fully reproduce the chronic course, lesion heterogeneity, microbial ecology, mucus properties, immune complexity, or treatment background of human IBD. Future studies should therefore prioritize mucosal pharmacokinetics, disease-site accumulation, dose–exposure relationships, standardized head-to-head comparisons, long-term oral safety, formulation reproducibility, scalability, and clinical validation.

## 6. Preclinical and Clinical Evidence Supporting the Therapeutic Potential of Berberine in Inflammatory Bowel Disease

### 6.1. Preclinical Evidence

A growing body of experimental evidence supports the protective effects of berberine in animal models of IBD. Commonly used models, including dextran sulfate sodium (DSS)- and 2,4,6-trinitrobenzene sulfonic acid (TNBS)-induced colitis, reproduce key pathological features of intestinal inflammation, including mucosal inflammation, epithelial injury, and immune disturbance [68,80,83]. Across these models, berberine administration has been associated with reduced disease activity index scores, alleviated histopathological injury, partial restoration of colon length, decreased inflammatory mediator production, and improved indices of mucosal integrity [129,150]. Together, these findings provide convergent support for the protective effects of berberine in experimental colitis.

This broad pattern of improvement suggests that berberine affects several pathological dimensions of experimental colitis, including inflammatory activation, epithelial injury, and mucosal barrier dysfunction [36,76,104]. However, most available data are derived from chemically induced colitis models, which do not fully reproduce the chronicity, biological heterogeneity, and complex host–environment interactions of human IBD. Therefore, while preclinical findings provide a strong rationale for further investigation, caution is needed when extrapolating these results directly to clinical disease. To clarify the pharmacological basis of berberine in UC, selected preclinical studies investigating its therapeutic effects, experimental models, dosing regimens, and molecular mechanisms are summarized in Table 2.

### 6.2. Clinical Evidence and Translational Perspectives

Compared with the substantial preclinical literature, clinical evidence supporting the use of berberine in IBD remains limited and is concentrated mainly on ulcerative colitis rather than Crohn’s disease. Experimental studies suggest that berberine may exert microbiota-modulating, anti-inflammatory, epithelial barrier-protective, and metabolic regulatory effects. However, whether these mechanisms translate into reproducible and clinically meaningful benefits in patients remains insufficiently established. Current interpretation should therefore distinguish mechanistic plausibility, preliminary human signals, and definitive therapeutic efficacy.

The most direct published human evidence comes from a small double-blind phase I trial in Chinese patients with biopsy-proven UC [151]. Sixteen patients who were in clinical remission or had minimal disease activity while receiving mesalamine maintenance therapy were randomized in a 3:1 ratio to receive oral berberine at 900 mg/day or placebo for 3 months, with 12 patients assigned to berberine and 4 to placebo. Berberine was generally well tolerated, although one grade 3 transaminase elevation and one grade 1 nausea event were reported among treated patients. Berberine also produced measurable plasma exposure and significantly reduced the Geboes histological grade in colonic tissue, suggesting a potential mucosal anti-inflammatory signal. However, most other inflammatory or cell-growth-related biomarkers were not significantly altered, and the trial was not designed to evaluate berberine as induction therapy for active moderate-to-severe UC. Thus, this study supports berberine as a preliminary adjunctive candidate to mesalamine, but not as definitive evidence of clinical efficacy. A related UC maintenance trial registered on ClinicalTrials.gov, entitled “Efficacy of Treatment With Berberine to Maintain Remission in Ulcerative Colitis” (NCT02962245), was withdrawn and therefore provides no efficacy or safety results.

Additional clinical evidence has been summarized in a recent systematic review and meta-analysis evaluating berberine combined with 5-aminosalicylic acid for UC [152]. This analysis included 10 randomized controlled trials (RCTs) involving 952 patients and reported improvements in clinical efficacy rate, Baron endoscopic score, disease activity index score, clinical symptoms, and inflammatory markers, without a significant increase in adverse reactions. Nevertheless, the certainty and generalizability of these findings remain limited. The included RCTs were mainly identified from Chinese-language literature and databases, which may limit international visibility, independent verification, and external generalizability. In addition, heterogeneity in patient populations, berberine formulations, dosing regimens, treatment duration, concomitant therapies, and outcome definitions may have influenced the pooled estimates. The commonly reported “clinical efficacy rate” is also not equivalent to contemporary international UC trial endpoints, such as clinical remission, endoscopic improvement, histological response or remission, corticosteroid-free remission, fecal calprotectin normalization, or C-reactive protein normalization.

Overall, berberine remains a promising but preliminary adjunctive candidate for UC, especially in combination with 5-aminosalicylic acid. At present, its therapeutic rationale in IBD relies mainly on preclinical evidence, supported by limited human studies and meta-analytic findings of variable quality. Key translational questions remain unresolved, including the optimal dose, treatment duration, long-term safety, local intestinal exposure, and patient populations most likely to benefit. These issues highlight the need for large, multicenter, randomized, double-blind, placebo-controlled trials using standardized berberine formulations, predefined dosing regimens, stratified patient populations, and clinically meaningful clinical, endoscopic, histological, biomarker, pharmacokinetic, and safety endpoints.

## 7. Challenges and Future Perspectives

Despite growing evidence for the therapeutic potential of berberine in IBD, several mechanistic and translational challenges remain. A key unresolved issue is the causal hierarchy within the microbiota–immune–barrier axis. Berberine has been reported to reshape the gut microbiota, reduce mucosal inflammation, and improve epithelial barrier function; however, it remains unclear whether microbial remodeling represents a primary driver of therapeutic efficacy, a secondary consequence of inflammation resolution, or part of a reciprocal regulatory loop. The temporal links among microbial metabolites, immune recalibration, and barrier repair also require clarification. Germ-free models, fecal microbiota transplantation, metabolomics, single-cell sequencing, and spatial transcriptomics may help define the causal sequence and tissue-specific mechanisms of berberine action.

Another major limitation is the reliance on preclinical colitis models. DSS-induced colitis mainly reflects acute epithelial injury, whereas TNBS-induced colitis is biased toward Th1-mediated inflammation. Although these models are useful for mechanistic studies, they do not fully reproduce the chronic, relapsing, and heterogeneous nature of human IBD. Chronic colitis models, humanized microbiota systems, patient-derived organoids, and longitudinal human cohorts are therefore needed to improve translational relevance.

Clinical translation also remains insufficient. Current human evidence is limited, and well-designed randomized controlled trials are needed to define efficacy, dose range, treatment duration, safety window, and clinically meaningful endpoints. Dose translation is particularly challenging because many animal studies use high doses that cannot be directly converted to humans by body weight alone. Body surface area-based human equivalent dose estimation may provide an initial reference, but it cannot replace formal pharmacokinetic, pharmacodynamic, dose-escalation, and safety assessments. Given berberine’s low systemic bioavailability and extensive intestinal metabolism, future studies should evaluate not only plasma exposure but also local luminal and mucosal exposure, which may be more relevant for microbiota- and mucosa-targeted effects. Long-term safety, tolerability, and potential drug–drug interactions also require systematic assessment, especially for adjunctive or maintenance use.

Patient heterogeneity represents another important barrier. Because berberine partly acts through the gut microbiota and microbial metabolites, baseline microbial composition may influence treatment response. Disease subtype, inflammatory activity, concomitant medication, diet, and metabolic status may also affect efficacy. Integrating metagenomics, metabolomics, host transcriptomics, and clinical phenotyping may help identify response biomarkers and support microbiota-guided patient stratification.

Delivery optimization should also move beyond improving solubility, stability, or oral absorption. Future systems should be designed to strengthen the interaction between berberine and key interfaces of the microbiota–immune–barrier axis. Microbiota-responsive formulations may promote local release at diseased intestinal sites, macrophage-targeted systems may enhance mucosal immunomodulation, and mucus-penetrating or bioadhesive carriers may support epithelial repair. However, these strategies still require evaluation of safety, reproducibility, manufacturability, scalability, and clinical feasibility.

Overall, future research should move from isolated mechanistic observations toward causal validation, clinically relevant models, standardized trials, and precision therapeutic strategies. Berberine should therefore be investigated not only as a conventional anti-inflammatory natural product, but also as a systems-level modulator of the intestinal ecosystem. Clarifying its mechanisms, responders, optimal exposure, and translational boundaries will be essential for advancing berberine-based strategies in IBD.

## 8. Conclusions

Berberine has emerged as a promising adjunctive candidate for IBD therapy, with therapeutic potential closely linked to coordinated regulation of the microbiota–immune–barrier axis. Current evidence suggests that berberine may modulate gut microbial composition and metabolism, attenuate mucosal immune activation, and support epithelial barrier restoration. This integrative mode of action is particularly relevant to IBD, a disease characterized by complex pathogenesis, mucosal ecosystem disruption, and heterogeneous treatment responses. However, most supporting evidence remains preclinical, and available clinical data are still preliminary.

Future progress will require causal mechanistic validation, clinically relevant models, optimized gut-localized formulations, and well-controlled clinical trials with standardized endpoints. These efforts will be essential to determine whether berberine can be translated from an experimental multi-target compound into a microbiota- and barrier-oriented therapeutic strategy for personalized IBD management.

## Figures and Tables

**Table 1:** Representative berberine delivery systems for gut-localized therapy in experimental IBD.

Strategy Category	Delivery System	Targeting Mechanism	Experimental Model	Main Advantages	Key Limitations	Reference
Colon-targeted release	Berberine-loaded pH-responsive Eudragit^®^ FS 30D-attapulgite microspheres	pH-triggered colonic release	In vitro simulated GI release; DSS-induced colitis mice	Enhanced colonic release; improved colitis symptoms; increased gut microbiota diversity and reduced inflammatory cytokines	pH-dependent release may vary under active inflammation	[55]
Colon-targeted release	pH/enzyme- or pH/microbiota-responsive berberine carriers, including BBR-ES microparticles and fructan-based berberine nanoparticles	Dual-responsive release triggered by colonic pH and microbial activity	In vitro release assays; DSS-induced colitis mice	Improved colonic delivery; reduced colitis severity and inflammatory cytokines; reshaped gut microbiota; increased tight junction protein expression in fructan-based nanoparticles	Efficacy may vary with microbiota composition and enzyme activity	[66,73]
Inflammation- and cell-responsive delivery	Berberine-loaded β-glucan nanoparticles	β-glucan-mediated macrophage uptake	LPS-induced RAW264.7 macrophages; DSS-induced C57BL/6 mouse UC model	Enhanced berberine uptake by macrophages; reduced IL-1β, IL-6, COX-2, and nitric oxide production; alleviated DSS-induced colitis	Macrophage targeting may vary with macrophage phenotype and inflammatory stage	[115]
Inflammation- and cell-responsive delivery	Berberine-loaded PLGA nanoparticles	Regulation of the macrophage–epithelial interface through the IL-6/IL-6R axis	DSS-induced UC mice; LPS-induced THP-1 macrophages; macrophage–NCM460 co-culture system	Improved berberine dispersibility and bioactivity; reduced M1 macrophage infiltration and IL-6 signaling; improved epithelial apoptosis and barrier function	Long-term oral safety, biodistribution, and off-target effects require further validation	[121]
Mucosal retention and barrier repair	Berberine-loaded chitosan/fucoidan-taurine complex nanoparticles	Local delivery to protect epithelial tight-junction barrier under inflammatory stimulation	Caco-2/RAW264.7 co-culture system	Protected tight-junction integ rity; improved TEER; reduced paracellular permeability; restored ZO-1 distribution under inflammatory injury	Evidence is mainly based on an in vitro epithelial–macrophage co-culture model; in vivo efficacy requires further validation	[144]
Mucosal retention and barrier repair	Berberine-containing gallic acid/pectin in situ hydrogel	Intestinal enzyme-triggered in situ gelation and mucoadhesive local retention	In vitro adhesion assays; in vivo intestinal retention/distribution; DSS-induced acute colitis in C57BL/6 mice	Enhanced local intestinal retention; reduced inflammation and oxidative stress; restored immune and microbiota homeostasis; strengthened epithelial barrier	Gel formation and adhesion may vary with intestinal enzyme activity, mucus turnover, and luminal fluid conditions	[148]

**Table 2:** Preclinical studies of berberine in ulcerative colitis: models, dosing regimens, therapeutic effects and mechanisms.

Experimental Model/Animal	Dose & Route	Treatment Duration	Key Findings	Proposed Mechanisms	Reference
3% DSS-induced UC in 8-week-old male BALB/c mice	BBR, 100 or 300 mg/kg, oral gavage daily	7 or 14 days; 100 mg/kg for 14 days was optimal	Reduced DAI, colonic injury and pro-inflammatory cytokines; increased IL-10, TGF-β, ZO-1 and Occludin	Modulation of gut microbiota and metabolites via the PDGFA/lithocholate sulfate/Alistipes axis	[46]
3% DSS-induced acute colitis in 6-week-old male BALB/c mice	BBR, 40 mg/kg/day, oral gavage	7 days	Reduced weight loss, DAI score, colon shortening and histological injury; restored intestinal mucosal barrier and immune homeostasis	Microbiota-dependent protection of mucosal barrier via activation of the Wnt/β-catenin pathway	[71]
2.5% DSS-induced acute colitis in 8-week-old male C57BL/6 mice	BBR, 100 mg/kg, oral administration	DSS for 10 days; BBR continued for 4–7 days after DSS	Reduced body weight loss, DAI, colon shortening, fecal LCN-2 and IgA; improved mucosal barrier integrity	Restored mucus barrier homeostasis and regulated mucin-degrading microbiota, especially Akkermansia and Bacteroides, promoting mucin–SCFA metabolism	[83]
Chronic DSS-induced UC in C57BL/6J mice	CCP 15 mg/kg, BBR 50 mg/kg, or CCP + BBR; oral administration	10 days	CCP + BBR showed stronger protection than either alone, improving colon pathology, inflammation, and tight-junction protein expression	Increased SCFA-producing bacteria and SCFA levels; activated AhR/IL-22 pathway	[90]
2.5% DSS-induced UC in 8-week-old male C57BL/6 mice	BBR, 10 or 50 mg/kg, oral gavage daily	7 days	Reduced body weight loss, DAI, colon shortening, serum TNF-α, IL-1β and IL-6, and mucosal injury	Modulated gut microbiome and bile acid metabolism in the gut–liver axis; restored barrier function via the bile acid/S1PR2/RhoA/ROCK pathway	[91]
DSS-induced colitis in male Sprague Dawley rats	BBR, 40 mg/kg, intragastric/oral gavage	7 days	Improved colitis symptoms, colon inflammation, gut barrier disruption and microbiota dysbiosis	Regulated gut microbiota-derived tryptophan metabolites and activated AhR signaling to restore intestinal barrier function	[92]
3% DSS-induced UC in mice; LPS-induced NCM460 cell model for in vitro validation	BBR, 100 mg/kg/day, oral gavage	DSS for 7 days; BBR from day 4 for 7 days	Reduced DAI, weight loss and intestinal inflammation; decreased IL-6 and TNF-α	Inhibited TLR4/NF-κB/HIF-1α signaling, reducing inflammatory responses and improving colonic pathology	[95]
2% DSS-induced colitis in male C57BL/6 mice; LPS-induced Caco-2 cells	BBR, 20/40/80 mg/kg, intragastric gavage; 5–20 μM in Caco-2 cells	7 days in vivo; 24 h in vitro	Reduced weight loss, DAI, colon shortening and histological injury; restored goblet cells and tight-junction proteins	Targeted/downregulated HSP90AA1 and MAPK14, reducing TNF-α and repairing intestinal mucosal barrier	[101]
LPS-induced murine intestinal organoids and RAW264.7 macrophage–organoid co-culture model	BBR, 1 μM in vitro	24 h	Restored organoid viability/budding, reduced inflammatory cytokines, inhibited macrophage chemotaxis and M1 polarization, and restored barrier proteins	Disrupted pathological macrophage–epithelial crosstalk; reduced CXCL1/CXCL2/CXCL5, PTGS2, CRP and IL-1β-related inflammatory signaling	[104]
DSS-induced UC mice with dampness-heat syndrome; LPS-induced RAW264.7 cells	BBR, 25/50/100 mg/kg; route not clearly shown in accessible preview	Drug intervention from days 28–34; 7 days	Improved UC symptoms, reduced inflammation and enhanced intestinal barrier protein expression	Directly targeted IRGM1 and inhibited PI3K/AKT/mTOR signaling	[108]

## Data Availability

No new data were created or analyzed in this study.

## Supplementary Materials

The following supporting information can be downloaded at: https://www.mdpi.com/article/10.3390/ijms27125220/s1.

## Abbreviations

The following abbreviations are used in this manuscript:

AA	Arachidonic acid
AhR	Aryl hydrocarbon receptor
AKT	Protein kinase B
AMPK	AMP-activated protein kinase
CD	Crohn’s disease
DSS	Dextran sulfate sodium
ERK	Extracellular signal-regulated kinase
FXR	Farnesoid X receptor
HIF-1α	Hypoxia-inducible factor-1 alpha
IBD	Inflammatory bowel disease
IFN-γ	Interferon gamma
IL	Interleukin
ILC2	Group 2 innate lymphoid cells
IRGM1	Immunity-related GTPase family M protein 1
JAK	Janus kinase
JNK	c-Jun N-terminal kinase
MAPK	Mitogen-activated protein kinase
MPO	Myeloperoxidase
mTOR	Mechanistic target of rapamycin
NF-κB	Nuclear factor kappa B
NLRP3	NOD-like receptor family pyrin domain containing 3
PI3K	Phosphoinositide 3-kinase
PLGA	Poly(lactic-co-glycolic acid)
QPCT	Glutaminyl-peptide cyclotransferase
ROS	Reactive oxygen species
SCFAs	Short-chain fatty acids
SOCS1	Suppressor of cytokine signaling 1
TGR5	G protein-coupled bile acid receptor 1
Th17	T helper 17
TLR	Toll-like receptor
TLR4	Toll-like receptor 4
TNBS	2,4,6-Trinitrobenzene sulfonic acid
TNF-α	Tumor necrosis factor alpha
Treg	Regulatory T cell
UC	Ulcerative colitis
ZO-1	Zonula occludens-1
