# Supplementary material for: Berberine in Inflammatory Bowel Disease: Integrative Regulation of the Microbiota–Immune–Barrier Axis
Source: Int J Mol Sci. 2026 Jun 9;27(12):5220. doi: 10.3390/ijms27125220 (PMC13299313; doi:10.3390/ijms27125220)
Supplement: Supplementary file 1 [file ijms-27-05220-s001.zip › ijms-4308427-supplementary.pdf]

Supplementary Table S1. Evidence hierarchy and representative quantitative or experimental support for proposed direct targets and indirectly modulated pathways of berberine

Target/pathway	Evidence category	Representative quantitative or experimental evidence	Interpretation in this review	References
NEK7/NLRP3 inflammasome	Direct target evidence	Berberine has been reported to directly target NEK7 and inhibit NEK7 activity with an IC50 of 4.2 μM. It also blocks the NEK7–NLRP3 interaction and suppresses inflammasome activation.	This represents comparatively stronger evidence for direct target engagement and provides a representative quantitative example supporting the multitarget potential of berberine. However, the evidence is not exclusively IBD-specific.	[51,52]
PLA2/COX-2/PGE2/ EP2 axis	Pathway-level validation; direct target engagement not established in IBD models	In DSS-induced UC models, berberine was reported to regulate the PLA2–COX-2–PGE2–EP2 inflammatory pathway. Direct PLA2 binding or potency data should be discussed only when supported by target-identification literature.	This supports modulation of inflammatory lipid-mediator signaling in UC models. In the absence of direct binding, KD, Ki, or IC50 data in the cited IBD models, this axis should be interpreted as pathway-level evidence rather than definitive direct target engagement.	[52,67];
TLR4/NF-κB/HIF-1α axis	In silico-supported and experimentally validated pathway evidence	Network pharmacology, molecular docking, and experimental validation support involvement of the TLR4/NF-κB/HIF-1α axis in berberine-mediated protection against UC-related inflammation.	This provides in silico-supported and experimentally validated pathway evidence, but does not by itself prove direct binding of berberine to TLR4, NF-κB, or HIF-1α.	[93]
NF-κB inflammatory signaling	In vitro/in vivo pathway validation	Berberine suppresses NF-κB activation and reduces inflammatory mediator production in TNBS- or DSS-induced colitis and inflammation-related cellular models.	This represents well-supported anti-inflammatory pathway modulation rather than evidence for a single direct molecular target.	[78,98,114]
Macrophage polarization-related pathways	Cellular and in vivo immune validation	Berberine inhibits M1 macrophage polarization through the AKT1/SOCS1/NF-κB axis and promotes M2-related responses through IL-4/STAT6-associated signaling in colitis models.	This supports immunomodulatory activity in intestinal inflammation and should be interpreted as regulation of immune-cell function rather than direct binding to all listed pathway proteins.	[112]
Th17/Treg immune balance	In vivo immune validation	Berberine regulates Th17/Treg imbalance and suppresses Th17-associated inflammatory responses in experimental colitis models.	This supports adaptive immune regulation in IBD-related inflammation, but does not indicate a defined direct molecular target.	[68,108]
Gut microbiota and	Microbiota-mediated	Berberine reshapes gut microbiota	This indicates indirect host–	[74,87,89,90]

microbial metabolites	functional evidence	and modulates microbial metabolites, including SCFAs, bile acids, and tryptophan-derived metabolites, in UC/colitis models.	microbiota-mediated mechanisms contributing to anti-inflammatory and barrier-protective effects.	
AhR activation	Microbiota-metabolite-mediated pathway validation	Berberine promotes AhR activation through microbial tryptophan catabolites in colitis models; related studies also support AhR/IL-22-associated mucosal protection.	This supports an indirect microbiota–metabolite–host signaling mechanism rather than direct AhR agonism unless direct binding data are provided.	[88,90]
Wnt/β-catenin signaling	In vivo epithelial repair validation	Berberine improves intestinal mucosal barrier dysfunction through microbiota-dependent Wnt/β-catenin signaling in DSS-induced colitis.	This supports epithelial repair and mucosal healing as downstream functional effects.	[69]
AMPK/MLCK and tight-junction regulation	Barrier-function validation	Berberine attenuates tight-junction injury and improves epithelial barrier integrity through AMPK/MLCK-related regulation and epithelial barrier models.	This supports barrier-protective effects in UC-related models, but should be interpreted as pathway modulation rather than direct target evidence.	[81,130,133]
HSP90AA1/MAPK14 and other predicted targets	Network pharmacology plus experimental association	Predicted targets such as HSP90AA1 and MAPK14 have been associated with berberine-mediated intestinal mucosal barrier repair.	These should be regarded as predicted and experimentally associated pathway targets, not definitive direct targets unless direct binding, KD, Ki, or IC50 data are reported.	[99]

Table note. Quantitative values are provided where available from the cited literature. Direct target evidence refers to studies reporting direct binding, enzymatic inhibition, biophysical validation, or quantitative potency values such as IC50, Ki, or KD. In silico-supported evidence refers to network pharmacology or molecular docking studies supported by experimental validation. Cellular or in vivo pathway validation refers to pathway modulation observed in experimental models without confirmed direct target engagement. Microbiota-mediated evidence refers to mechanisms primarily supported by changes in gut microbiota, microbial metabolites, or microbiota-dependent host signaling. Absence of quantitative potency values for a given pathway indicates that the mechanism is supported by pathway modulation, cellular or in vivo outcomes, or microbiota-associated changes rather than confirmed direct target engagement. Therefore, the targets and pathways summarized in this table should not be interpreted as equivalent direct molecular targets of berberine.
